# *Trichophyton indotineae*, an Emerging Drug-Resistant Dermatophyte: A Review of the Treatment Options

**DOI:** 10.3390/jcm13123558

**Published:** 2024-06-18

**Authors:** Benedetta Sonego, Andrea Corio, Vanessa Mazzoletti, Verena Zerbato, Alessandro Benini, Nicola di Meo, Iris Zalaudek, Giuseppe Stinco, Enzo Errichetti, Enrico Zelin

**Affiliations:** 1 Dermatology Clinic, Maggiore Hospital, University of Trieste, 34125 Trieste, Italy; benedettasong@gmail.com (B.S.); andrea.corio.fe@gmail.com (A.C.); beninialessandro1996@gmail.com (A.B.); nickdimeo@libero.it (N.d.M.); iris.zalaudek@gmail.com (I.Z.); 2 Institute of Dermatology, Santa Maria della Misericordia University Hospital, 33100 Udine, Italy; vanessa.mazzoletti@gmail.com; 3 Infectious Diseases Unit, Trieste University Hospital (ASUGI), 34125 Trieste, Italy; verena.zerbato@gmail.com; 4 Institute of Dermatology, Department of Medicine, University of Udine, 33100 Udine, Italy; giuseppe.stinco@uniud.it (G.S.); enzoerri@yahoo.it (E.E.)

**Keywords:** *Trichophyton indotineae*, tinea, drug resistance

## Abstract

**Background:** Dermatophytosis is a prevalent superficial infection caused by filamentous fungi, primarily affecting the skin and/or its appendages. In recent years, there has been a notable increase in mycotic strains resistant to standard antifungal therapies, including *Trichophyton indotineae*, a dermatophyte of the *Trichophyton mentagrophytes* complex. This review aims to provide a comprehensive overview of the treatment options for *T. indotineae*, elucidating their effectiveness in managing this challenging mycotic infection. **Methods:** For this review, a search was conducted in the PubMed, Scopus, Web of Science, Embase, and Google Scholar databases, encompassing all published data until March 2024. English-language articles detailing therapy outcomes for patients confirmed to be affected by *T. indotineae*, identified through molecular analysis, were included. **Results:** Itraconazole was shown to be a good therapeutic choice, particularly when administered at a dosage of 200 mg/day for 1–12 weeks. Voriconazole was also demonstrated to be effective, while terbinafine exhibited a reduced response rate. Griseofulvin and fluconazole, on the other hand, were found to be ineffective. Although topical treatments were mostly ineffective when used alone, they showed promising results when used in combination with systemic therapy. Mutational status was associated with different profiles of treatment response, suggesting the need for a more tailored approach. **Conclusions:** When managing *T. indotineae* infections, it is necessary to optimize therapy to mitigate resistances and relapse. Combining in vitro antifungal susceptibility testing with mutational analysis could be a promising strategy in refining treatment selection.

## 1. Background

Dermatophytosis is a superficial fungal infection that can involve skin, hair, and nails. It is caused by dermatophytes, filamentous fungi belonging to the genera *Trichophyton*, *Microsporum*, and *Epidermophyton*, which survive by consuming keratin [1,2]. Variants of dermatophytosis affecting the epidermis include tinea corporis (trunk), tinea cruris (groin), tinea faciei (face), tinea manuum (hands), and tinea pedis (feet). Infections involving the hair follicle can result in tinea capitis (scalp) and tinea barbae (beard). Lastly, fungal nail infection is referred to as tinea unguium or onychomycosis [1,2]. Dermatophytosis is highly prevalent, affecting about 20–25% of the global population [1,2]. Diagnosis relies on clinical appearance and can be confirmed by identifying hyphae in fresh potassium hydroxide (KOH) preparations of skin scale samples or through culture examinations of skin scales, hairs and/or nails [3].

## 2. Antimycotic Drugs: Basic Principles

Azoles and allylamines represent two groups of widely used antimycotic drugs, available in topical and oral formulations. Itraconazole, fluconazole, and voriconazole are triazoles, while terbinafine is an allylamine. On the other hand, griseofulvin does not belong to these categories, since it has a different mechanism of action [4].

Itraconazole and other synthetic triazoles act by inhibiting the fungal cytochrome P450-dependent enzyme lanosterol 14-α-demethylase. This inhibition prevents the conversion of lanosterol to ergosterol, thereby disrupting fungal cell membrane synthesis [5]. Notably, systemic itraconazole is currently available in both conventional and super bioavailable (SUBA) formulations. SUBA formulation improves bioavailability by employing a pH-dependent polymeric matrix that releases the drug in the proximal small intestine, where absorption occurs. This approach differs from the conventional one, which relies on dissolution and absorption primarily in the stomach [6]. SUBA-itraconazole provides more consistent serum levels with less inter-patient variation, eliminating the need for an acidic environment for dissolution and overcoming food-related variability [7]. Studies showed that SUBA-itraconazole has a relative bioavailability of 173% compared to conventional itraconazole, suggesting that 58 mg of SUBA-itraconazole provides exposure equivalent to 100 mg of the conventional form [8].

Terbinafine, like other allylamines, operates by blocking the squalene epoxidase enzyme (SQLE) controlled by the *ERG1* gene. This action inhibits ergosterol production in fungal cell membranes. Consequently, squalene accumulates, inducing disruption of the fungal cell membrane and ultimately eliciting fungicidal effects [9].

Finally, griseofulvin, derived through industrial fermentation of the fungus *Penicillium griseofulvum*, acts by binding to fungal microtubules, thereby disrupting their function and inhibiting mitosis [10].

## 3. Treatment of Dermatophytosis and Emergence of Antifungal Resistance

Depending on severity and extent, dermatophytosis can be treated with a wide range of topical and oral antifungal drugs, either as monotherapy or combination therapy.

The majority of tinea corporis cases can be effectively managed solely with topical therapy, which should be the preferred option whenever feasible, due to its safer profile and minimal side effects. Topical allylamines (such as terbinafine and naftifine) and topical azoles (including clotrimazole, bifonazole, sulconazole, miconazole, sertaconazole, eberconazole, econazole, oxiconazole, and luliconazole) are all viable options for treating dermatophytosis [11]. However, topical allylamines, particularly terbinafine, have demonstrated higher cure rates and shorter treatment durations compared to topical azoles [12].

In cases of extensive infections or inadequate response to topical treatments, systemic therapy becomes necessary. Commonly prescribed oral treatments include itraconazole (50–200 mg/day), fluconazole (50 mg/day–150 mg/week), terbinafine (250 mg/day), and griseofulvin (500–1000 mg/day), with results typically expected within a few weeks [13,14]. Randomized controlled trials have underscored their efficacy, particularly noting better outcomes for terbinafine and itraconazole compared to griseofulvin [15,16].

For severe infections, oral systemic therapy can be supplemented with topical antifungal treatments. A recent review suggests that combining antifungal agents may enhance both clinical and microbiological healing of a superficial infection, potentially expediting the recovery process [17].

In clinical practice, fixed-drug combinations (FDCs) containing corticosteroids alongside antifungal agents are also widely used. While corticosteroids offer rapid symptom relief by mitigating local inflammatory reactions, their inclusion may promote improper self-medication [18,19,20]. Incorrect and uncontrolled use of FDCs and topical antifungals may contribute to the emergence and spread of multidrug-resistant dermatophytosis [21,22]. Moreover, after the wide use of oral terbinafine for dermatophytosis due to its safety and pharmacokinetic profile, resistance to this drug has begun to emerge. Clinical studies have shown reduced efficacy with standard dosing regimens (250 mg/day), leading to empirical use of higher doses and longer durations [23,24,25,26].

Microbiological resistance is defined as the fungus’s inability to respond to an antifungal agent, as evidenced by in vitro antifungal susceptibility testing (AFST). Microbiological resistance can be intrinsic or acquired. The latter, which develops among previously susceptible strains, typically occurs after exposure to antifungal agents and is often a result of altered gene expression. AFST determines the minimum inhibitory concentration (MIC) of antifungal drug required to inhibit the growth of a dermatophyte in vitro. To date, established breakpoints to assess dermatophyte resistance to specific antimycotic agents are lacking. Notably, a limitation of this approach is that MICs may not consistently predict clinical response [27,28].

Dermatophyte infections are increasingly demonstrating resistance to standard antifungal treatments worldwide. Dermatologists in India have raised awareness about the prevalence of recalcitrant widespread dermatophytoses [29,30] and in recent years, *Trichophyton indotineae*, a member of the *Trichophyton mentagrophytes* complex, has been identified as an emerging resistant mycotic strain [31].

## 4. *Trichophyton indotineae* Epidemiology

*Trichophyton indotineae* (also named *Trichophyton mentagrophytes* ITS genotype VIII) was identified as a new dermatophyte species in 2020, in India [32]. *T. indotineae* stands out as a distinct species due to its unique internal transcribed spacer (ITS) region sequencing, as well as mycological and physiological features [33].

*Trichophyton rubrum* (known to mostly cause tinea corporis, tinea pedis, and tinea unguium) used to be the major agent causing dermatophytosis in India, as well as worldwide, but is now being replaced by agents of the *T. mentagrophytes* complex [2,29]. Compared to other pathogens of the same genera, *T. indotineae* is more commonly associated with chronic and relapsing infections. Differently from *T. rubrum*, most cases of *T. indotineae* infection present with highly inflammatory lesions accompanied by itching and a burning sensation, often involving the lower part of the body and the inguinal region, causing tinea corporis, cruris, and, less frequently, faciei and pedis [2,29]. *T. indotineae* often exhibits resistance to terbinafine due to mutations in the squalene epoxidase gene. In recent times, strains resistant to azoles are emerging [34,35]. The aforementioned use of over-the-counter corticosteroid–antifungal combinations may have contributed to the spread of terbinafine-resistant *Trichophyton* strains in India [19].

Although *T. indotineae* is emerging significantly as a cause of dermatophytosis throughout the Indian subcontinent, mycoses caused by this strain have been documented beyond the borders of India, spreading to Asia, the Middle East, Europe and America [33].

Most cases identified outside the Indian subcontinent are related to travel or migration. Cases have been described in China [36,37], Kuwait [38], Japan [39], Vietnam [40], the United Arab Emirates [41], Turkey [42], Iran [43], and Singapore [44]. In the United States (US), at the beginning of 2023, two cases of tinea unresponsive to oral terbinafine treatment were reported, subsequently identified as being caused by *T. indotineae*. Interestingly, one of the two patients had no recent international travel history [45]. In the early months of 2024, in the US, a case of tinea of the genital region caused by *T. indotineae* and potentially sexually transmitted was described [46]. Cañete-Gibas et al., however, demonstrated that the presence of *T. indotineae* strains in the US dates back to at least 2017 [47]. A case series of eight patients also indicated the presence of *T. indotineae* in Canada [48]. Messina et al. recently reported the first case of tinea corporis caused by *T. indotineae* in Latin America, specifically in Argentina, occurring in a patient with a previous 8-month stay in Mexico [49].

Regarding Europe, *T. indotineae* isolations are spreading in many countries: Denmark [50], Belgium [51], Greece [52], Switzerland [53,54], Spain [55], France [56], the United Kingdom [57], and Germany [58]. In Germany, genotype analysis has shown that certain *Trichophyton spp*. strains dating back to 2011 are compatible with *T. indotineae* [59]. In Italy, the first patients with *T. indotineae* infections were reported in 2023. Only one of them had not traveled outside Italy in the previous years; the others had traveled from Pakistan, Egypt, and India [60,61].

## 5. Resistance Mechanisms in *T. indotineae*

Resistance mechanisms in dermatophytes can be attributed to various factors, including overexpression of drug efflux pumps and formation of biofilm. However, one of the most commonly reported mechanisms in *T. indotineae* involves point mutations in the squalene epoxidase (*SQLE*) gene, *ERG1*, which have been associated with terbinafine resistance. These mutations ultimately alter terbinafine binding to the SQLE enzyme [27].

Compared to the wild-type, *T. indotineae* isolates with Phe397Leu and Leu393Ser SQLE mutations demonstrate reduced susceptibility to terbinafine in vitro [27]. Conversely, the Ala448Thr mutant exhibits higher susceptibility to terbinafine, although it is subject to debate whether this mutant may be less sensitive to azoles [62,63]. The double mutation Phe397Leu/Ala448Thr is linked to decreased susceptibility to terbinafine and azoles [64]. Similarly, double mutant Leu393Ser/Ala448Thr shows reduced susceptibility to terbinafine, as well [27].

SQLE mutations are significant and well-known contributors to antifungal resistance in *T. indotineae*. However, resistance to azoles has also been recently observed in mutants with upregulation of ABC transporters or mutation in *ERG11/CYP51* gene. The *ERG11* gene, which is the target of azoles, encodes the lanosterol 14-α-demethylase enzyme involved in the ergosterol biosynthesis pathway [27,62].

Of note, Gupta et al. pointed out the potential usefulness of combining antifungal susceptibility testing with mutational analysis: the presence of the aforementioned mutations, along with elevated MICs, suggests an increased risk of clinical resistance to antimycotic drugs (such as terbinafine or azoles), which warrants further investigation [27].

Efforts are currently underway to develop diagnostic tools for resistant *T. indotineae* infections and to explore therapeutic alternatives. Therefore, we conducted a literature review to collect data on the reported treatment options for *T. indotineae* and their effectiveness.

## 6. Methods

A comprehensive literature search was conducted, including all published data up to March 2024, across multiple databases (PubMed, Scopus, Web of Science, Embase, and Google Scholar). The search terms utilized were ‘*Trichophyton indotineae*’ OR ‘*Trichophyton mentagrophytes* ITS genotype VIII’ OR ‘*Trichophyton mentagrophytes* ITS type VIII’ OR ‘*Trichophyton mentagrophytes* complex’. Additionally, potentially relevant papers were identified by manually checking the references of the included literature.

All selected articles underwent a full-text assessment to determine their eligibility. Inclusion criteria comprised patients diagnosed with *T. indotineae* infection confirmed by molecular identification, presenting tinea corporis, cruris, faciei, or pedis. Tinea capitis, barbae, and unguium were excluded, ensuring a consistent population. Only articles detailing therapy with dosage and duration, and reporting treatment outcomes, were included. Furthermore, only articles written in English were considered. The literature search and eligibility assessment were conducted independently by A.C. and B.S., and any disagreement was discussed between them.

## 7. Results

After the selection process, 25 articles met the inclusion criteria, describing a total of 58 patients affected by tinea caused by *T. indotineae*, confirmed by molecular analysis. Sometimes, multiple cycles of therapy were carried out in the same patient (with different molecules or dosages). Therefore, in this analysis, the total number of treatment instances (*n* = 126) exceeded the overall number of patients.

From a clinical standpoint, the majority of patients presented with a widespread form of tinea corporis treated with numerous topical and systemic treatments for several weeks without success. Additionally, nine patients with tinea cruris, one with tinea pedis, and one with tinea faciei were also collected [35,39,42,43,46]. Very rarely, complete resolution was achieved with the first therapeutic attempt (six cases) [53,56,59,61,65].

Data on *T. indotineae* cases, treatments, and outcomes are reported in Table 1.

## 8. Oral Itraconazole

According to our analysis, itraconazole emerged as the most prescribed treatment for *T. indotineae* and showed a good response rate. Dosages varied from 50 mg twice daily (BID) of SUBA-itraconazole up to 400 mg/day of traditional itraconazole, with treatment duration ranging from 1 to 12 weeks. In some cases, pulse therapy was adopted, involving daily drug administration for one week every month, in a variable period of time.

A total of 49 instances of *T. indotineae* treated with itraconazole were collected. Among them, 22 cases achieved complete resolution, 4 experienced improvement, 4 had resolution followed by relapse, 5 showed improvement followed by disease recurrence, and 14 had no response to treatment.

The most effective treatment leading to disease resolution was itraconazole at a dosage of 200 mg/day for a variable duration of 1 to 12 weeks, with 15 described cases [41,55,57,58,60,65,67,68,69]. The majority of patients (six cases) were cured with itraconazole 200 mg/day for 2 weeks [60,67]. However, cases of itraconazole failure at the same dosage were also reported. For example, Bhattacharyya et al. described seven cases of tinea corporis/cruris not responsive to itraconazole 200 mg/day for 8 weeks [35].

Regarding lower doses of itraconazole, complete healing has been described with SUBA-itraconazole at a dosage of 50 mg BID (100 mg/day) for 4 weeks [49]. Disease resolution has also been documented with itraconazole at a dosage of 100 mg/day for a duration of 4 weeks [43,48]. Moreover, Fattahi et al. reported three cases of itraconazole pulse therapy (100 mg/day for one week each month for 3 or 5 months) resulting in improvement but subsequent disease relapse [43].

Higher doses of itraconazole, such as 400 mg/day, demonstrate similar efficacy compared to the 200 mg/day regimen [56]. Spivack et al. documented a case of tinea cruris cured with itraconazole 200 mg BID (400 mg/day) for 3 weeks [46]. Kong et al. also described resolution achieved with itraconazole 200 mg BID (400 mg/day) for 5 weeks [56,70]. Meanwhile, Fukada et al. reported a case of tinea faciei that was resolved with itraconazole 400/day mg for one week, following an initial failure with a reduced dosage of itraconazole 100 mg/day for 4 weeks [39]. Nenoff et al. described a case of tinea cruris treated with complete resolution with itraconazole 400 mg/day for 8 weeks [58]. Finally, a case of tinea pedis achieved resolution and then relapsed, despite itraconazole pulse therapy with 400 mg/day administered 1 week/month for 2 months [43].

In general, itraconazole appeared to be relatively effective, also showing a synergistic effect with topical azoles, particularly clotrimazole cream, ketoconazole 2% cream, luliconazole 1% cream, and bifonazole cream [40,41,65,69].

## 9. Oral Voriconazole

All five selected cases treated with voriconazole responded positively to therapy, achieving complete resolution [38,43,66]. The dosages described were 200 mg/day for 2 weeks in one patient [66], 200 mg/day for 4 weeks in two patients [43], and 400 mg BID (800 mg/day) for 2 days followed by 200 mg BID (400 mg/day) for 3 months in one patient [38]. Additionally, Fattahi et al. described a case of tinea pedis resolved with voriconazole at a dosage of 50 mg/day for 2 weeks [43].

## 10. Oral Fluconazole

We identified four patients treated with fluconazole, and none of them were cured. Three of them showed no response to treatment (fluconazole 50 mg/day for 10 days in combination with hydrocortisone and clotrimazole ointment, fluconazole 150 mg/day for 20 days combined with sertaconazole cream, and fluconazole 150 mg/day for 11 weeks, respectively) [43,66].

In contrast, an improvement followed by relapse was observed with a dosage of fluconazole 200 mg/week for 16 weeks associated with tobramycin topical treatment [69].

## 11. Oral Ketoconazole

The usage of ketoconazole was described in only one patient. Dosage of 200 mg BID (400 mg/day) for 6 weeks without associated topical treatment showed improvement but no complete healing [66].

## 12. Oral Terbinafine

Among the 44 selected cases of *T. indotineae* treated with terbinafine, dosages varied from 125 mg/day to 250 BID (500 mg/day), with treatment duration ranging from 10 days to 24 weeks.

Only five cases achieved clinical resolution (at a dosage of 250 mg/day for a treatment duration ranging from 4 to 12 weeks) [53,56,59,61]. Associated topical treatments that favored disease resolution included ciclopirox, terbinafine, and ketoconazole cream applied throughout the duration of oral therapy [53,56,59].

In nine instances, improvement with terbinafine at a dosage of 250 mg/day was achieved (for 4, 8, and 9 weeks, respectively) [56,60,69].

Two patients showed clinical improvement during therapy, but later experienced recurrence of the disease. One patient was treated with terbinafine 250 mg/day combined with terbinafine cream for 2 weeks, while the second one received terbinafine 250/day for 3 weeks without topical treatment [56,70].

In twenty-eight cases there was no improvement with terbinafine (125–500 mg/day, with a variable duration ranging from 10 days to 24 weeks) [35,38,42,43,46,48,55,56,60,66,67,68,69,70,71]. In general, a treatment duration exceeding 12 weeks with terbinafine 250 mg/day did not demonstrate greater efficacy [42,56,71].

## 13. Oral Griseofulvin

Of the five patients who received oral griseofulvin, none experienced resolution of the disease, thus defining it as an ineffective treatment [38,56,69,71]. Even when administered at a dosage of 1000 mg/day for 24 weeks, it did not demonstrate efficacy [56]. Furthermore, combining it with topical therapy (econazole cream) did not result in improvement [71].

## 14. Topical Treatment

In most cases, topical treatment has proven efficacy only when combined with systemic therapy.

The use of topical ketoconazole has been described in two cases. The first patient had an extensive tinea corporis and applied ketoconazole cream for 12 weeks, in combination with terbinafine 250 mg/day [53]. Additionally, Ngo et al. reported resolution with itraconazole 200 mg/day for one week and ketoconazole 2% cream for two weeks [40].

Regarding clotrimazole, Pavlovic et al. described a case resolved with the combination of itraconazole 200 mg/day and clotrimazole cream for 8 weeks [41], while Kong et al. successfully combined a twice-daily application of clotrimazole cream with itraconazole 200 mg BID (400 mg/day) for 5 weeks [70].

Thakur et al. reported a case successfully treated with luliconazole cream 1% in combination with itraconazole 200 mg/day for 8 weeks [65].

The use of topical bifonazole combined with itraconazole 200 mg/day for 12 weeks also demonstrated efficacy, with complete clearance of the cutaneous mycosis [69].

Moreover, a case of therapeutic success with the combination of topical terbinafine and oral terbinafine 250 mg/day for 4 weeks was described [56].

As for the use of topical treatment alone, without associated systemic treatment, only three cases of clinical resolution have been reported. Among these, Gueneau et al. described healing with the use of topical voriconazole 1% applied for 8 weeks [71]. The patient had previously been treated for many weeks with oral terbinafine and griseofulvin associated with topical terbinafine, econazole, terbinafine, and bifonazole, without improvement. Moreover, Jabet et al. described a case of resolution through the application of ciclopirox olamine cream for 4 weeks in the absence of oral treatment [56]. Additionally, Nenoff et al. described a case of extensive dermatophytosis resolved with a combination of ciclopirox olamine cream and topical miconazole, for 4 weeks [58].

Finally, topical treatment with omoconazole 2% cream for 4 weeks followed by miconazole for 4 weeks resulted in clinical improvement but no complete healing [56,58]. Additionally, bifonazole 1% cream for 3 weeks demonstrated temporary improvement with subsequent disease relapse [56].

## 15. Mutational Profile and Associated Effectiveness of Therapy

Among the 25 identified studies, mutational analysis of the *SQLE* gene was performed in 15 studies, covering a total of 36 patients.

In five out of thirty-six cases (13.9%), no mutations associated with terbinafine resistance were detected: four patients carried wild-type SQLE and one carried a three-mutation SQLE variant (A3360G; G3606T; A3734G), characterized by a behavior similar to the wild-type enzyme [40,56,61,67]. All these patients were cured, responding to oral terbinafine (3/5) [56,61], oral itraconazole (1/5) [67], and oral itraconazole plus topical ketoconazole (1/5, three-mutation variant) [40].

Phe397Leu mutation was identified in 16 out of 36 patients (44.4%) [38,42,43,56,58,60,69,70]. Among them, 12 patients achieved a complete response, mostly with azoles; specifically, oral itraconazole was effective in 7 out of the 12 patients, voriconazole in 4 out of 12, and topical miconazole and ciclopirox olamine in 1 out of 12 [38,43,58,60,69,70]. Moreover, improvement was reported in some cases with the use of oral itraconazole and oral terbinafine [42,43,58,69,70].

Ala448Thr mutation was detected in 6 out of 36 patients (16.7%) [53,56,58,59,69]. In this group, four patients had a complete response: one with oral itraconazole, one with oral terbinafine, one with oral terbinafine plus topical ketoconazole, and one with topical ciclopirox olamine [53,56,58,59], while 2 out of 6 patients showed solely an improvement (with oral fluconazole plus topical tobramycin, and oral terbinafine, respectively) [69].

Leu393Ser mutation was found in 4 out of 36 patients (11.1%) [56,60,71]. Among them, two out of four had a complete response (one with oral itraconazole and one with topical voriconazole) [60,71], while the remaining two out of four showed only mild improvement (one with topical bifonazole, one with oral terbinafine) [56].

One single case (1 out of 36, 2.8%) had Phe415Cys mutation, which was detected and registered by Bortoluzzi et al. [60]. This mutant was successfully treated with oral itraconazole [60].

Double substitution in the *SQLE* gene was found in three out of thirty-six patients (8.3%), with two cases of Phe397Leu/Ala448Thr mutation [53,56] and one case of Phe397Leu/Thr414His [42]. None of them was cured; only one of the Phe397Leu/Ala448Thr mutants showed mild improvement after treatment with oral itraconazole [56].

Finally, among the known mutants collected in this review, the case of Bhattacharyya et al. stands out (1 out of 36, 2.8%): in fact, this specific dermatophyte carried mutations in SQLE (Phe397Leu), *ERG11*, *ERG4*, *MDR1*, and *MFS* genes, along with a novel *ERG3* mutation. It is worth noting that *ERG11*, *ERG3* and *ERG4* are involved in the ergosterol synthesis pathway, while *MDR1* and *MFS* genes are part of the ABC transporter superfamily [35]. From a clinical standpoint, it failed a sequential course of oral treatment with terbinafine and itraconazole [35].

Data on mutations and respective treatment regimens are shown in Table 2.

## 16. Discussion

The emergence of *Trichophyton indotineae* has significantly transformed the treatment landscape for dermatophytosis. Once easily managed with short-term antifungal therapy, mycotic infections can now become chronic and recurrent, requiring prolonged treatment due to increasing antifungal resistance.

Itraconazole is demonstrated to be a good first-line choice for *T. indotineae* infections. While previous literature has reported no significant difference in outcome between 100 and 200 mg/day dosages, our review revealed a different scenario [72]. In fact, our analysis clearly indicates that a dosage of 100 mg/day, regardless of the therapy duration, was generally insufficient to obtain complete cure, even for prolonged therapies (up to eight weeks) [43,48]. On the other hand, disease resolution was achieved most effectively with itraconazole 200 mg/day, administered for a variable duration ranging from 1 to 12 weeks, as evidenced by 15 reported cases [41,55,57,58,60,65,67,68,69].

Some studies have explored itraconazole serum levels in patients treated for tinea corporis or cruris. Despite higher serum levels of itraconazole being found in those taking higher doses, there is no correlation between serum levels and treatment response [73,74,75].

Although the best response rate was obtained with itraconazole 200 mg/day for 2 weeks (in a total of six cases) [60,67], it is essential to underline the fact that some cases required prolonged treatment lasting 4, 6, 8, and 12 weeks, to achieve complete resolution [41,55,57,58,60,65,67,68,69]. These data illustrate the challenge of standardizing treatment in terms of dosage and duration, emphasizing the need for personalized approaches: therapy duration should be adjusted based on the patient’s response, continuing until resolution. In some cases, a two-week treatment suffices, while in others, extension up to twelve weeks may be necessary (particularly for diffuse forms of tinea corporis), although longer therapy duration entails potentially greater side effects and costs [55,69].

Of note, in our review we included only one case treated with SUBA-itraconazole, which resulted in complete resolution at a dosage of 50 mg BID for 4 weeks [49]. The expected clinical response to SUBA-itraconazole closely resembles that of conventional itraconazole when administered at equivalent doses.

According to our analysis, in the azoles group, oral voriconazole was also demonstrated to be very effective in treating *T. indotineae* dermatophytosis, achieving complete resolution in all instances. The reported dosages and treatment durations varied, mainly consisting of 200 mg/day for 2–4 weeks [38,43,66]. Despite the excellent results highlighted in these papers, voriconazole is described in the literature as a poorly effective drug for dermatophytosis (in particular for tinea corporis and cruris), burdened by high costs and significant risk of resistance development [72]. Further investigation through clinical trials is warranted for its use in treating *T. indotineae*.

Oral fluconazole was found to be ineffective for *T. indotineae,* with none of the treated patients experiencing disease resolution or significant clinical improvement, even when administered at various dosages and treatment durations. Possible explanations for the low efficacy of fluconazole include its weak affinity for keratin and rapid elimination from the stratum corneum within a timeframe of about 60 to 90 h [76]. With this premise, weekly intake seems not advisable, as there is no depot effect to sustain the pharmacologic action after discontinuation [72]. These reasons lead to considering fluconazole as inadequate in the therapeutic management of *T. indotineae*.

Similarly, oral ketoconazole exhibited limited efficacy, with improvement observed in one case but no cases of complete healing [66]. Not only is ketoconazole burdened by many side effects, but it also fails to provide any benefits in terms of effectiveness, treatment duration, or rates of recurrence, compared to terbinafine and itraconazole.

In our review, oral terbinafine exhibited limited efficacy in treating *T. indotineae* infections, with only a small number of cases achieving clinical resolution (250 mg/day for 4–12 weeks). Treatment was more effective when combined with topical antifungal agents such as ciclopirox, ketoconazole, or terbinafine creams [53,56,59]. Of note, longer treatment durations exceeding 12 weeks did not demonstrate greater efficacy [42,56,71]. Terbinafine ineffectiveness could be attributed to the rising resistance associated with the widespread use of this drug. Moreover, most SQLE mutations in *T. indotineae* are ultimately linked to reduced terbinafine susceptibility, while this antifungal maintains a potential role in wild-type SQLE cases [56,61], as well as in SQLE Ala448Thr mutants [53,59]. Further exploration of this topic will be conducted in the following sections.

Unfortunately, griseofulvin has been shown to be ineffective in all described cases, even at different dosages and durations. Our data align with the literature; in fact, griseofulvin has been associated with poor outcomes, mainly for its scarce adhesion to keratin and the washout effect caused by sweating [76,77].

According to our review, topical treatment alone was largely ineffective in the majority of the described cases, showing better results in conjunction with systemic therapies. The topical treatments that demonstrated higher effectiveness and promoted complete healing in combination with systemic therapy were ketoconazole, clotrimazole, luliconazole and ciclopirox olamine.

Ketoconazole cream showed efficacy in combination with both terbinafine and itraconazole [40,53]. Similarly, topical clotrimazole [41,70] and topical luliconazole [65] resulted in complete remission only when associated with systemic itraconazole. Ciclopirox olamine cream was effective, either when combined with terbinafine [59] or as a single treatment [56,58].

Although other topical therapies have sporadically shown positive effects in combination with systemic treatment, these are typically long-term treatments, ranging from a minimum of 4 up to 12 continuous weeks [53,56,58,71].

It is assumed that topical treatment acts as an adjunct in the treatment of cutaneous mycosis, thus requiring an associated systemic treatment to achieve complete resolution of the clinical condition. Isolated cases of complete healing were also reported with the use of topical voriconazole or ciclopirox olamine cream alone, indicating potential effectiveness in selected cases [56,58,71].

In general, it is debated whether the association of a topical treatment with an oral antifungal therapy could lead to a better outcome compared to systemic therapy alone, because large-scale studies focusing on this question are lacking.

As far as mutational status is concerned, it is known that antifungal sensitivity of *T. indotineae* varies according to its mutational profile, particularly regarding the *SQLE* gene.

Based on the data of this review, the majority of wild-type SQLE patients (3 out of 4) were successfully treated with oral terbinafine [56,61]. However, one case did not respond to this drug but healed with oral itraconazole [67]. Another case described by Ngo et al. (triple mutation A3360G, G3606T, A3734G of *SQLE* gene) can be assimilated to the wild-type SQLE group: it did not show lower in vitro sensitivity to terbinafine and responded to oral itraconazole plus topical ketoconazole [40].

Among the SQLE Phe397Leu mutants, all patients who achieved complete response (12 cases) were treated with oral azoles; of them, 7 were cured with oral itraconazole. None of the patients healed with terbinafine [38,43,58,60,69,70]. Among the eight patients with the same mutation who experienced improvement, seven were treated with oral itraconazole, and just two were treated with oral terbinafine [42,43,58,69,70]. These observations seem to confirm that Phe397Leu mutants tend to show lower sensitivity to terbinafine, in vitro just as in the clinical setting [27].

On the other hand, the six patients with SQLE Ala448Thr mutation improved or healed completely, thanks to oral terbinafine in 3/6 cases [53,59,69], while oral azoles and ciclopirox olamine cream proved effective as well [56,58,69]. This seems to confirm that Ala448Thr mutants are in general responders to terbinafine, while in the literature it is debated whether this mutation could be associated with lower sensitivity to azoles [62,63].

Regarding the four patients with the SQLE Leu393Ser mutation, three out of four responded to treatment with azoles (two cases of resolution and one case of improvement), while terbinafine proved to be ineffective [56,60,71]. These findings are consistent with the evidence suggesting that Leu393Ser mutants appear less sensitive to terbinafine both in vitro and in the clinical setting [27].

The SQLE Phe415Cys mutant, detected and registered by Bortoluzzi et al., did not respond to terbinafine but showed a complete response to itraconazole. This clinical result reflects the MIC values indicating lower in vitro susceptibility to terbinafine but high susceptibility to itraconazole. From a molecular standpoint, the Phe415Cys mutation is located in pivotal positions for the binding of terbinafine to the SQLE enzyme, which may justify this behavior [60].

Concerning the SQLE double mutation Phe397Leu/Ala448Thr, one case improved thanks to treatment with oral itraconazole, while oral treatments with terbinafine and griseofulvin did not lead to a clinical response [56]. Another case was treated with topical econazole and ketoconazole, with no improvement [53]. These variants are particularly challenging to treat, being associated with a lower sensitivity to terbinafine and increased resistance to azoles [64].

Regarding the SQLE double mutation Phe397Leu/Thr414His reported by Durdu et al., it remains unclear if it enhances antifungal resistance, but the patient did not improve with terbinafine, temporarily responding to fluconazole but relapsing after treatment [42].

In addition to SQLE mutations leading to terbinafine resistance, upregulation of ABC transporters or mutation in *ERG11/CYP51* gene has been associated with resistance to azoles [27]. The patient, reported by Bhattacharyya et al., carrying Phe397Leu SQLE mutation and *ERG11B* mutations, did not respond to oral therapy with terbinafine or itraconazole; it should be noted that this case also had mutations in the *ERG3*, *ERG4*, *MDR1* and *MFS* genes [35]. This particular scenario should raise concern about the spreading of *T. indotineae* resistant to both terbinafine and itraconazole, considering that azoles are the main therapeutic choice in case of allylamine treatment failure [27].

Facing increasing fungal resistances, new strategies are essential. Recent hypotheses focus on innovative solutions, such as combining different antifungals or associating antifungals with retinoids.

While systemic antifungal combination therapies for *T. indotineae* are yet to be explored in clinical practice, their use may lead to complications such as drug interactions, increased adverse effects and higher costs [78]. Validating synergistic antifungal combinations necessitates rigorous in vitro testing but also well-designed clinical trials [79]. Despite promising in vitro findings with some combinations, their efficacy in a real-life context remains uncertain [78], and recent studies failed to demonstrate significant advantages over monotherapy [80,81].

On the other hand, the association of antifungals (in particular, itraconazole) with retinoids shows potential benefits. Retinoids enhance cell proliferation, theoretically impeding the spread of ongoing dermatophytosis and decreasing the recurrence rate [82]; additionally, they increase skin pH and boost humoral and cellular immunity [83]. Although data on the combination of retinoids and antifungals are still scarce to date, it would appear that the combination of itraconazole and low-dose isotretinoin may have a positive impact on cases of recalcitrant tinea, leading to earlier complete cure and a significant reduction in recurrence rates [84,85]. However, Verma et al. found that adding isotretinoin to terbinafine did not improve treatment outcomes for recurrent dermatophytosis and was associated with several retinoid-related side effects [86].

In summary, further investigations are needed to understand the relationship between in vitro microbial resistance and in vivo clinical resistance regarding *T. indotineae*. However, based on the literature and the results of our review, we argue that the combination of antifungal susceptibility testing and SQLE mutation detection could help determine the most effective treatment for patients affected by *T. indotineae*. Finally, the effectiveness and safety profile of combination therapies for recalcitrant forms of tinea in a real-life scenario is still a matter of debate.

Limitations of our review include the low number of cases, as our inclusion criteria required articles with precise therapy details, thus excluding cases with unspecified treatment regimens. The scarcity of literature concerning certain drug categories such as fluconazole and voriconazole is evident, making it difficult to conduct a fair comparison between various treatments due to different sample sizes. Moreover, the selected articles consist primarily of case reports, which represent the lowest level of scientific evidence. In addition, there is a risk of reporting bias, as case reports may not always provide a comprehensive representation of the treatment outcomes, and ineffective therapies could be under-reported. Finally, mutational analysis was conducted only in a small portion of cases.

## 17. Conclusions

*T. indotineae* infections, initially observed in India, are now reported worldwide and pose a growing problem, manifesting as recalcitrant tinea corporis and cruris. Considering treatment costs and post-cure relapse rates, therapy decisions must be weighed carefully.

Systemic azoles represent the primary treatment for *T. indotineae* infections, with oral itraconazole (at least 200 mg/day) being a reasonable first-line choice, also for Phe397Leu or Leu393Ser SQLE mutants. Even though a two-week treatment can suffice, prolonged therapy up to 12 weeks may be necessary, and a tailored approach is advisable.

As for oral voriconazole, its efficacy varies and this antifungal agent should not be considered standard practice, given its importance in the treatment of invasive mycoses.

Conversely, terbinafine 250 mg/day with treatment durations of up to 12 weeks could still be useful in the presence of both wild-type SQLE or Ala448Thr SQLE mutation. However, resistance to this drug is rising.

Finally, there is insufficient evidence to support the routine use of griseofulvin and fluconazole.

The use of systemic combination therapies (different antifungals or association with retinoids) for *T. indotineae* is not backed by robust scientific evidence, highlighting the need for further research to validate synergistic combinations effective in real-life practice.

In this evolving landscape, combining antifungal susceptibility testing and molecular analysis for the detection of SQLE mutation could aid in determining the most effective therapy. Unfortunately, double mutants in SQLE and also emerging strains of *T. indotineae* bearing both SQLE and *ERG11* mutations still pose a therapeutic challenge, showing a reduced response to allylamine and azole treatments. Given the limited options and evidence available, there is a pressing need for optimizing treatment regimens and developing new therapeutic agents for *T. indotineae* infections.

## 18. Future Directions

There is still much to uncover regarding the mutations that potentially make *T. indotineae*, and mycotic strains in general, particularly resistant to the available therapies.

In essence, the future objective is to customize therapies as much as possible to offer a tailored treatment to each patient. In the case of recalcitrant dermatophytosis, standardizing the combination of in vitro antifungal susceptibility testing (AFST) and squalene epoxidase (SQLE) mutational analysis could potentially represent a winning strategy for accurate diagnosis and effective therapy. Additionally, investigating the efficacy and safety of new therapeutic protocols, including combination therapies, could improve treatment outcomes for *T. indotineae* infections.

## Figures and Tables

**Table 1:** Treatments prescribed for the management of dermatophytosis caused by *Trichophyton indotineae*. Where not otherwise specified in the notes, the diagnosis was tinea corporis. Abbreviations: *n* = number of instances; SUBA = super bioavailable; mg = milligrams; qd = quaque die (once a day); BID = bis in die (twice a day).

Oral Treatment	Topical Treatment	Outcome	N of Patients	Notes
Itraconazole (*n* = 49)				
SUBA-itraconazole 50 mg BID (100 mg/day) × 4 weeks [49]	No topical	Resolution	1	
Itraconazole 100 mg qd × 2 weeks [43,48]	No topical [43,48]	Improvement [43,48]	3	Tinea pedis (one case) [43]
	No topical [43,48]	No improvement [43,48]	1	
	Hydrocortisone powder 1% in ciclopirox cream × 2 weeks [43,48]	No improvement [43,48]	1	
Itraconazole 100 mg qd × 4 weeks [39,43,48]	No topical [39]	Resolution, then relapse [39]	1	Tinea faciei [39]
	No topical [48]	No improvement [48]	1	
	No topical [43,48]	Resolution [43,48]	2	
Itraconazole 100 mg qd × 8 weeks [66]	No topical	No improvement	1	
Itraconazole 100 mg BID (200 mg/day) × 4 weeks [42]	No topical [42]	Resolution, then relapse [42]	1	
Itraconazole 100 mg BID (200 mg/day) × 8 weeks [42]	No topical [42]	No improvement [42]	1	Tinea cruris [42]
Itraconazole 200 mg qd × 1 week [40,48]	Ketoconazole 2% cream [48]	No improvement [48]	1	
	Ketoconazole 2% cream × 2 weeks [40]	Resolution [40]	1	
Itraconazole 200 mg qd × 2 weeks [60,67]	No topical [60,67]	Resolution [60,67]	6	
Itraconazole 200 mg qd × 4 weeks [57,58]	No topical [57,58]	Resolution [57,58]	2	
Itraconazole 200 mg qd × 6 weeks [68]	No topical	Resolution	1	
Itraconazole 200 mg qd × 8 weeks [35,41,65,69]	No topical [35]	No improvement [35]	7	Tinea corporis/cruris [35]
	No topical [69]	Resolution [69]	1	
	Clotrimazole cream [41]	Resolution [41]	1	
	Luliconazole cream (1% *w*/*w*) × 8 weeks [65]	Resolution [65]	1	
Itraconazole 200 mg qd × 12 weeks [55,69]	No topical [69]	Improvement, then relapse [69]	2	
	Bifonazole × 12 weeks [69]	Resolution [69]	1	
	No topical [55]	Resolution [55]	1	
Itraconazole 200 mg BID (400 mg/day) × 3 weeks [46,70]	No topical [46]	Resolution [46]	1	Tinea cruris [46]
	Naftifine hydrochloride and ketoconazole cream × 3 weeks [70]	Resolution, then relapse [70]	1	
Itraconazole 200 mg BID (400 mg/day) × 4 weeks [38]	No topical	No improvement	1	
Itraconazole 200 mg BID (400 mg/day) × 5 weeks [70]	Clotrimazole cream twice daily × 5 weeks	Resolution	1	
Itraconazole 400 mg qd × 1 week [39]	No topical	Resolution	1	Tinea faciei [39]
Itraconazole 400 mg qd × 8 weeks [56,58]	No topical [56,58]	Improvement [56]	1	
		Resolution [58]	1	
Itraconazole pulse therapy 100 mg BID 1 week/month × 3 months [43]	No topical	Improvement, then relapse	1	
Itraconazole pulse therapy 100 mg BID 1 week/month × 5 months [43]	No topical	Improvement, then relapse	2	Tinea pedis (one case) [43]
Itraconazole pulse therapy 400 mg qd 1 week/month × 2 months [43]	No topical	Resolution, then relapse	1	Tinea pedis [43]
Voriconazole (*n* = 5)				
Voriconazole 50 mg qd × 2 weeks [43]	No topical	Resolution	1	Tinea pedis [43]
Voriconazole 200 mg qd × 2 weeks [66]	No topical	Resolution	1	
Voriconazole 200 mg qd × 4 weeks [43]	No topical	Resolution	2	
Voriconazole 400 mg BID (800 mg/day) × 2 days, then 200 mg BID (400 mg/day) × 3 months [38]	No topical	Resolution	1	
Fluconazole (*n* = 4)				
Fluconazole 50 mg qd × 10 days [43]	Hydrocortisone and clotrimazole ointment × 10 days	No improvement	1	Tinea pedis [43]
Fluconazole 150 mg qd × 20 days [43]	Sertaconazole cream	No improvement	1	
Fluconazole 150 mg qd × 11 weeks [66]	No topical	No improvement	1	
Fluconazole 200 mg/week × 16 weeks [69]	Tobramycin	Improvement, then relapse	1	
Ketoconazole (*n* = 1)				
Ketoconazole 200 mg BID (400 mg/day) × 6 weeks [66]	No topical	Improvement	1	
Terbinafine (*n* = 44)				
Terbinafine 125 mg qd × 2 weeks [67]	No topical	No improvement	1	
Terbinafine 250 mg qd × 10 days [70]	Ketoconazole cream BID × 10 days	No improvement	1	
Terbinafine 250 qd × 2 weeks [46,70]	Terbinafine cream × 2 weeks [70]	Improvement, then relapse [70]	1	
	No topical [46]	No improvement [46]	1	Tinea cruris [46]
Terbinafine 250 qd × 3 weeks [56]	No topical	Improvement, then relapse	1	
Terbinafine 250 mg qd × 4 weeks [43,48,56,59,60,68]	Ciclopirox cream × 4 weeks [59]	Resolution [59]	1	
	Sertaconazole cream × 4 weeks [43]	No improvement [43]	1	
	Topical terbinafine × 4 weeks [56]	Resolution [56]	1	
	Topical terbinafine × 40 days [60]	Improvement [60]	5	
	No topical [48,56,68]	Improvement [56]	1	
		No improvement [48,68]	2	
Terbinafine 250 mg qd × 40 days [43]	Sertaconazole cream × 2 weeks [43]	No improvement	1	
	Terbinafine ointment × 12 weeks [43]	No improvement	1	
Terbinafine 250 mg qd × 6 weeks [35,38]	No topical [35,38]	No improvement	8	Tinea corporis/cruris (seven cases) [35]
Terbinafine 250 mg qd × 8 weeks [55,56,69,71]	Topical terbinafine × 8 weeks [71]	No improvement	1	
	No topical [55,56,69]	No improvement [55,56,69]	3	
		Improvement [69]	1	
		Improvement [56]	1	
		Resolution [56]	1	
Terbinafine 250 mg qd × 9 weeks [69,71]	Bifonazole × 9 weeks [71]	No improvement [71]	1	
	No topical [69]	Improvement [69]	1	
Terbinafine 250 mg qd × 12 weeks [53,56,61,69]	No topical [56,69]	No improvement [56,69]	2	
	Ketoconazole × 12 weeks [53]	Resolution [53]	1	
	No topical [61]	Resolution [61]	1	Concomitant tinea unguium not considered in this review. Ciclopirox nail solution was also applied on nails × 12 weeks.
Terbinafine 250 mg qd × 13 weeks [71]	Topical terbinafine × 13 weeks	No improvement	1	
Terbinafine 250 mg qd × 24 weeks [42,56]	Topical terbinafine × 24 weeks [42]	No improvement [42]	2	Tinea cruris (one case) [42]
	No topical [56]	No improvement [56]	1	
Terbinafine 250 mg BID (500 mg/day) × 10 weeks [66]	No topical	No improvement	1	
Griseofulvin (*n* = 5)				
Griseofulvin 500 mg BID (1000 mg/day) × 4 weeks [69]	No topical	No improvement	1	
Griseofulvin 500 mg BID (1000 mg/day) × 9 weeks [71]	Econazole	No improvement	1	
Griseofulvin 1000 mg qd × 6 weeks [38]	No topical	No improvement	1	
Griseofulvin 1000 mg qd × 12 weeks [56]	No topical	No improvement	1	
Griseofulvin 1000 mg qd × 24 weeks [56]	No topical	No improvement	1	
Topical treatment only (*n* = 18)				
No oral	Terbinafine 1% [41]	No improvement	1	
No oral	Terbinafine tincture and naftifine hydrochloride & ketoconazole cream BID × 2 weeks [70]	No improvement	1	
No oral	Terbinafine 1% × 3 weeks [57]	No improvement	1	
No oral	Terbinafine cream × 8 weeks [58]	No improvement	1	
No oral	Ciclopirox olamine cream × 4 weeks [56]	Resolution	1	
No oral	Ciclopirox olamine cream, miconazole × 4 weeks [58]	Resolution	1	
No oral	Ciclopirox olamine cream, sertaconazole × 5–6 weeks [58]	No improvement	1	
No oral	Bifonazole 1% cream × 3 weeks [56]	Improvement, then relapse	1	
No oral	Bifonazole 1% cream × 8 weeks [69]	No improvement	1	
No oral	Clotrimazole 1% cream—betamethasone dipropionate × 2 weeks [57]	Improvement, then relapse	1	
No oral	Clotrimazole cream × 1 week [48]	No improvement	1	
No oral	Econazole 1% cream × 12 weeks [56]	No improvement	1	
No oral	Econazole 1% cream × 36 weeks [53]	No improvement	1	
No oral	Voriconazole 1% × 8 weeks [71]	Resolution	1	
No oral	Voriconazole 1% × 24 weeks [71]	Resolution, then relapse	1	
No oral	Ketoconazole × 12 weeks [53]	No improvement	1	
No oral	Miconazole × 4 weeks [56]	Improvement	1	
No oral	Omoconazole 2% cream × 4 weeks [56]	Improvement	1	

**Table 2:** Association between *Trichophyton indotineae* squalene epoxidase (*SQLE*) gene mutation and treatment that led to cure (complete response). Where not otherwise specified in the notes, the diagnosis was tinea corporis. Abbreviations: *n* = number of instances; SQLE = squalene epoxidase; mg = milligrams; qd = quaque die (once a day); BID = bis in die (twice a day).

SQLE Mutation (*n* = 36)	Treatment That Led to Cure	No. of Cured Patients	Notes
Wild-type (*n* = 4) Total no. of cured patients: 4/4 [56,61,67]	Itraconazole 200 mg qd × 2 weeks [67]	1	
	Terbinafine 250 mg qd × 8 weeks [56]	2	
	Terbinafine 250 mg qd × 12 weeks [61]	1	Concomitant tinea unguium not considered in this review. Ciclopirox nail solution was also applied on nails × 12 weeks.
Wild-type assimilated (*n* = 1) Total no. of cured patients: 1/1 [40]	Itraconazole 200 mg qd × 1 week + topical ketoconazole 2% × 2 weeks [40]	1	Three-point mutation (A3360G; G3606T; and A3734G) not associated with terbinafine resistance, hence considered by the authors a terbinafine-sensitive dermatophyte, as with wild-type cases [40].
Phe397Leu (*n* = 16) Total no. of cured patients: 12/16 [38,42,43,56,58,60,69,70].	Itraconazole 100 mg qd × 4 weeks [43]	1	
	Itraconazole 200 mg qd × 2 weeks [60]	2	
	Itraconazole 200 mg qd × 8 weeks [69]	1	
	Itraconazole 200 mg qd × 12 weeks + topical bifonazole × 12 weeks [70]	1	
	Itraconazole 400 mg qd × 8 weeks [58]	1	
	Voriconazole 50 mg qd × 2 weeks [43]	1	Tinea pedis [43]
	Voriconazole 200 mg qd × 4 weeks [43]	2	
	Voriconazole 400 mg BID (800 mg/day) × 2 days, then 200 mg BID (400 mg/day) × 3 months [38]	1	
	Topical miconazole and ciclopirox olamine × 4 weeks [58]	1	
	Itraconazole pulse therapy 200 mg BID (400 mg/day) × 5 weeks [70]	1	
Ala448Thr (*n* = 6) Total no. of cured patients: 4/6 [53,56,58,59,69]	Itraconazole 200 mg qd × 4 weeks [58]	1	
	Terbinafine 250 mg qd × 4 weeks [59]	1	
	Terbinafine 250 mg qd + topical Ketoconazole × 12 weeks [53]	1	
	Topical ciclopirox olamine cream × 4 weeks [56]	1	
Leu393Ser (*n* = 4) Total no. of cured patients: 2/4 [56,60,71]	Itraconazole 200 mg qd × 2 weeks [60]	1	
	Topical voriconazole 1% × 24 weeks [71]	1	The patient also had subsequent tinea capitis (excluded from the analysis), succesfully treated with topical voriconazole 1% × 8 weeks [71].
Phe415Cys (*n* = 1) Total no. of cured patients: 1/1 [60]	Itraconazole 200 mg qd × 2 weeks [60]	1	
Phe397Leu/Ala448Thr (*n* = 2) Total no. of cured patients 0/2 [53,56]	/	0	Improvement with oral itraconazole 400 mg qd × 8 weeks in one case [56].
Phe397Leu/Thr414His (*n* = 1) Total no. of cured patients 0/1 [42]	/	0	
Other (*n* = 1) Total no. of cured patients 0/1 [35]	/	0	Dermatophyte carrying mutations in SQLE (Phe397Leu), *ERG11*, *ERG3*, *ERG4*, *MDR1* and *MFS* genes [35].

## Data Availability

All available information is contained within the manuscript.
